# Prenatal characterization of a novel inverted *SMAD2* duplication by mate pair sequencing in a fetus with dextrocardia

**DOI:** 10.1002/ccr3.3608

**Published:** 2020-12-10

**Authors:** Cinthya J. Zepeda‐Mendoza, Anna Essendrup, Stephanie A. Smoley, Sarah H. Johnson, Nicole L Hoppman, George Vasmatzis, Daniel L. Jackson, Hutton M. Kearney, Linda B. Baughn

**Affiliations:** ^1^ Cytogenetics and Genomic Microarray Laboratory ARUP Laboratories Salt Lake City UT USA; ^2^ Division of Laboratory Genetics Department of Laboratory Medicine and Pathology, Mayo Clinic Rochester MN USA; ^3^ Center for Individualized Medicine‐Biomarker Discovery, Mayo Clinic Rochester MN USA; ^4^ Department of Molecular Medicine Mayo Clinic Rochester MN USA; ^5^ Department of Obstetrics, Gynecology and Women's Health University of Missouri Health Columbia MO USA

**Keywords:** mate pair, *SMAD2*, duplication, dextrocardia, heterotaxy

## Abstract

This case report underlines the importance of molecular characterization of genomic duplications and other structural variants in the prenatal setting to guide clinical interpretation, genetic counseling, and perinatal medical care.

## Graphical abstract

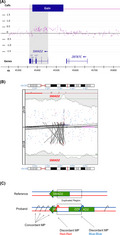

## 1 INTRODUCTION

A fetus harboring a duplication of *SMAD2* (exons 1‐6) presented with dextrocardia and pulmonary hypoplasia. Mate pair sequencing revealed the duplication to be in an inverted tandem orientation to the wild‐type *SMAD2* allele, disrupting its sequence and decreasing expression. These observations suggest *SMAD2* to be responsible for the fetal dextrocardia.

Accurate characterization of genomic structural variation is essential to prenatal genetic screening and clinical diagnosis of fetuses with abnormal ultrasound findings. Currently, prenatal genomic abnormalities ranging from aneuploidy to single‐nucleotide variants (SNVs) can be detected with a combination of genetic testing strategies, including but not limited to chromosome karyotyping, fluorescence *in situ* hybridization (FISH), chromosome microarray (CMA), and next‐generation sequencing (NGS).^1^, ^2^, ^3^, ^4^ CMA in particular has been shown to substantially increase prenatal diagnostic yields compared with chromosome banding analyses, given its ability to detect clinically relevant submicroscopic duplications and deletions (copy‐number variants, CNVs).^1^, ^5^ Chromosome deletions have been extensively associated with abnormal phenotypes through their impact on haploinsufficient gene expression, an observation that facilitates prenatal deletion classification and reporting. In contrast, fetal chromosome duplications have posed a more challenging and potentially uncertain interpretative context. At the functional level, duplications have the potential to result in overexpression of triplosensitive genes, disruption of haploinsufficient genes at their breakpoints, or creation of gene fusions, all of which could contribute to pathogenesis.^6^ The clinical utility of characterizing duplications by NGS to inform their mechanism of pathogenesis has been previously shown,^6^ with duplications in inverted orientation being more often associated with complex chromosome rearrangements.

Mate pair sequencing (MPseq) is an NGS technology specifically designed for the detection of genomic structural variants.^7^, ^8^ The method is based on the generation of large insert libraries (2‐5 kb), followed by paired‐end (PE) sequencing. Mapping of PE reads can easily reveal the presence of structural rearrangements with a high degree of confidence, including translocations, inversions, insertions, and copy‐number variants.^8^, ^9^, ^10^

In this report, we describe the MPseq analysis of a fetus with dextrocardia, pulmonary hypoplasia, and a complex conotruncal anomaly. Dextrocardia is a rare congenital condition in which the heart points toward the right side of the chest rather than the left^11^ and is frequently associated with heterotaxy, with important morbidity contributions due to abnormal pulmonary venous connection and ventricle obstructions.^12^ CMA performed on fetal amniotic fluid revealed a 145‐Kbp duplication at 18q21.1, including exons 1‐6 of the SMAD Family Member 2 (*SMAD2*) gene. While *SMAD2* plays an important role in mouse left‐right embryonic patterning^13^ and mutations have been linked to dextrocardia^14^ and other heart abnormalities in humans,^15^ the CMA results alone could not be used to determine whether the duplicated segment disrupted *SMAD2* gene function. MPseq was necessary to clarify the role of this duplication in the clinical phenotype of the fetus by revealing the duplicated segment to be in an inverted tandem orientation to the normal chromosome segment. This finding was predicted to disrupt *SMAD2* as evidenced by its reduced gene expression in cultured amniocytes.

## 2 RESULTS

### 2.1 Case presentation

**FIGURE 1:**
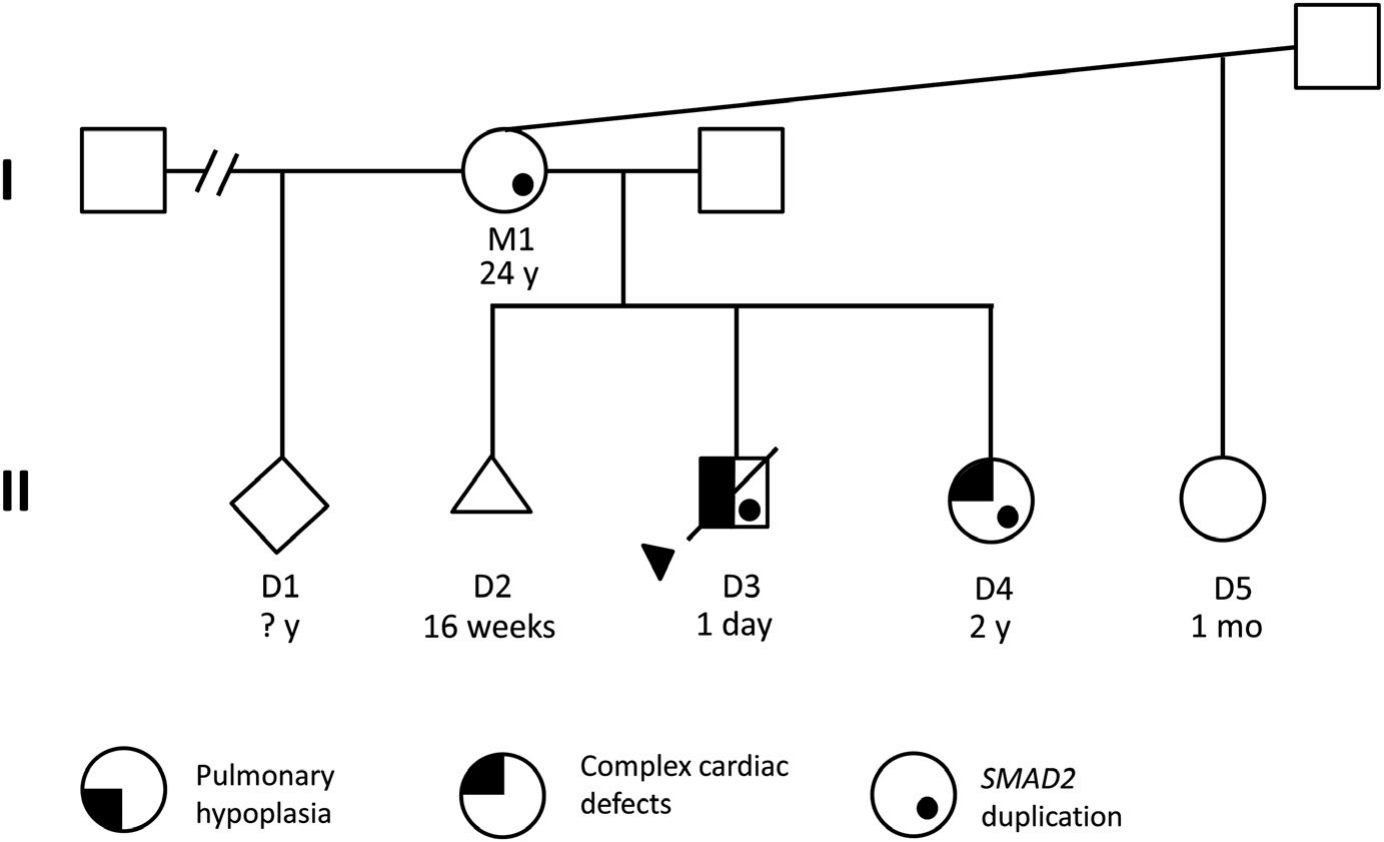
Pedigree showing segregation of a 145‐Kbp duplication at 18q21.1, including exons 1‐6 of *SMAD2*. Proband is indicated with a black arrow (D3). Cardiac anomalies in D3 include dextrocardia and conotruncal anomalies, while D4 presented with a single ventricle and TAPVR

A 22‐year‐old P3G4 woman (M1) was referred for genetic testing due to fetal anomalies detected by ultrasound. The fetus (the proband in this report, D3) was found to have dextrocardia, conotruncal anomalies, and pulmonary hypoplasia, and unfortunately passed away the day after delivery. Previous M1 pregnancies include a phenotypically normal child (D1) and one miscarriage (D2). During the writing of this report, M1 had two more pregnancies, delivering a child with complex cardiac defects, including a single ventricle and total anomalous pulmonary venous return (TAPVR) (D4), and another child (D5) who is alive and well at one month (Figure 1). M1 presents with sickle cell trait, but is otherwise reportedly healthy, without major surgeries or hospitalizations between pregnancies, and with no evidence of heterotaxy syndrome as revealed by a recent echocardiogram. Maternal and paternal families have no known consanguinity.

**FIGURE 2:**
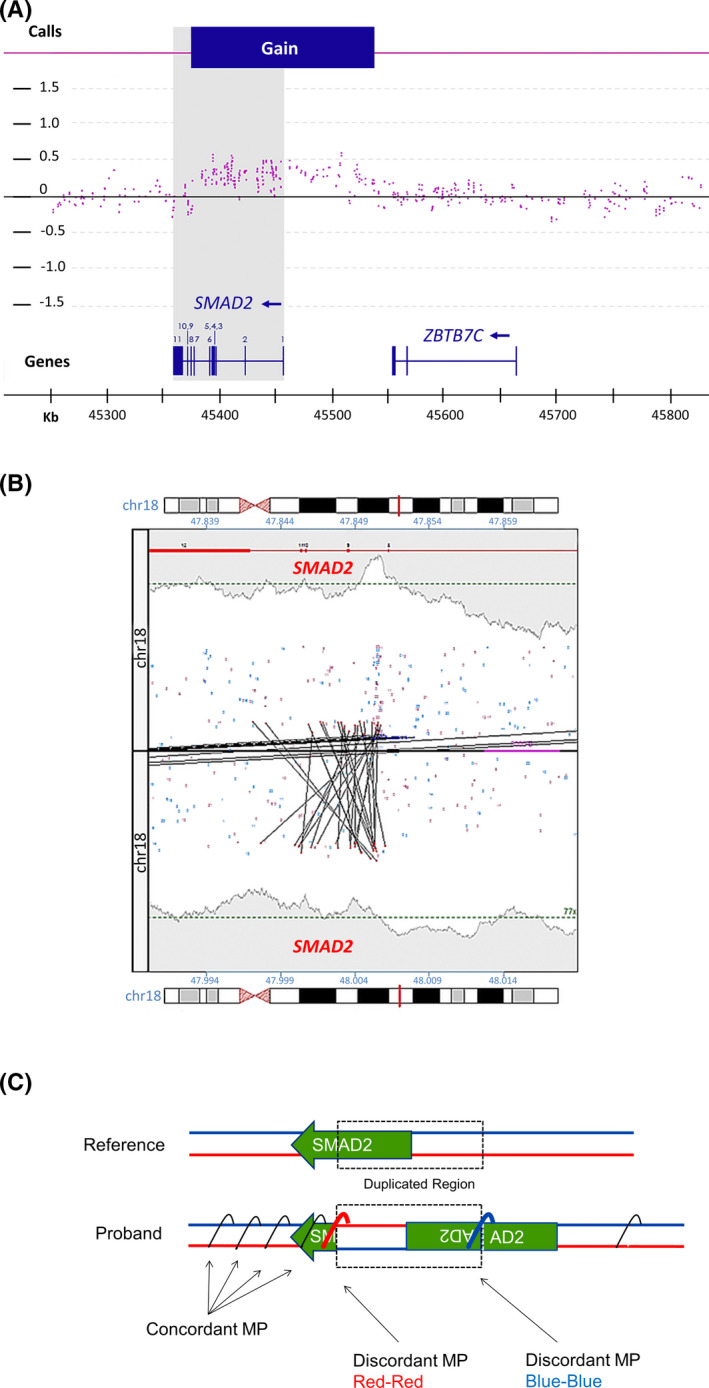
arr[GRCh37] 18q21.1(45384387_45529362)x3 mat duplication in proband. A, Screenshot of the duplication position as seen in ChAS software. The duplication is shown in the first horizontal panel as a blue rectangle. Probe positions are shown in the middle panel as dots. The elevated signal for probes directly below the blue rectangle were used for calling the duplication positions. Gene positions (*SMAD2* and *ZBTB7C*) are shown in the third panel, along with the nucleotide positions in chromosome 18. Exon numbers are displayed for *SMAD2*. The shaded rectangle encompasses *SMAD2* positions and shows the partial duplication. The 145‐Kbp duplication overlapped exons 1 through 6 of *SMAD2*, which could potentially disrupt the gene's function. In addition, the duplication was found to be maternally inherited in proband, but the mother was phenotypically normal. B, The *SMAD2* rearrangement as seen in our MPseq analysis pipeline. This junction plot displays four breakpoints representing the inversion event (colored dots indicate orientation of mapped reads, with blue corresponding to forward strand and red the reverse strand). The presence of the blue‐blue and red‐red dot associationsare predicted to be seen in a duplication with inverted orientation. Only discordant matepair reads with end positions mapping >15 Kbp are shown. C, Schematic representation of the *SMAD2* duplication seen in proband compared with the reference allele. Concordant and discordant matepair fragments and their corresponding orientations are shown in colored text

To characterize the etiology of the fetal dextrocardia found in proband (D3), genetic testing was performed on an amniocentesis sample from M1. Aneuploidy FISH analysis revealed a normal signal pattern for chromosomes X, Y, 13, 18, and 21 (Figure S1); however, CMAs revealed the presence of a duplication at 18q21.1 at g.47858016_48002991 (human genome version GRCh38). The duplication, approximately 145 Kbp in size, partially overlapped *SMAD2* (NM_005901) from exons 1 through 6 (Figure 2A). CMA studies on blood of the phenotypically unaffected mother (M1) revealed the duplication to be inherited. The same *SMAD2* duplication was subsequently found in proband’s affected sister (Figure 1).

**FIGURE 3:**
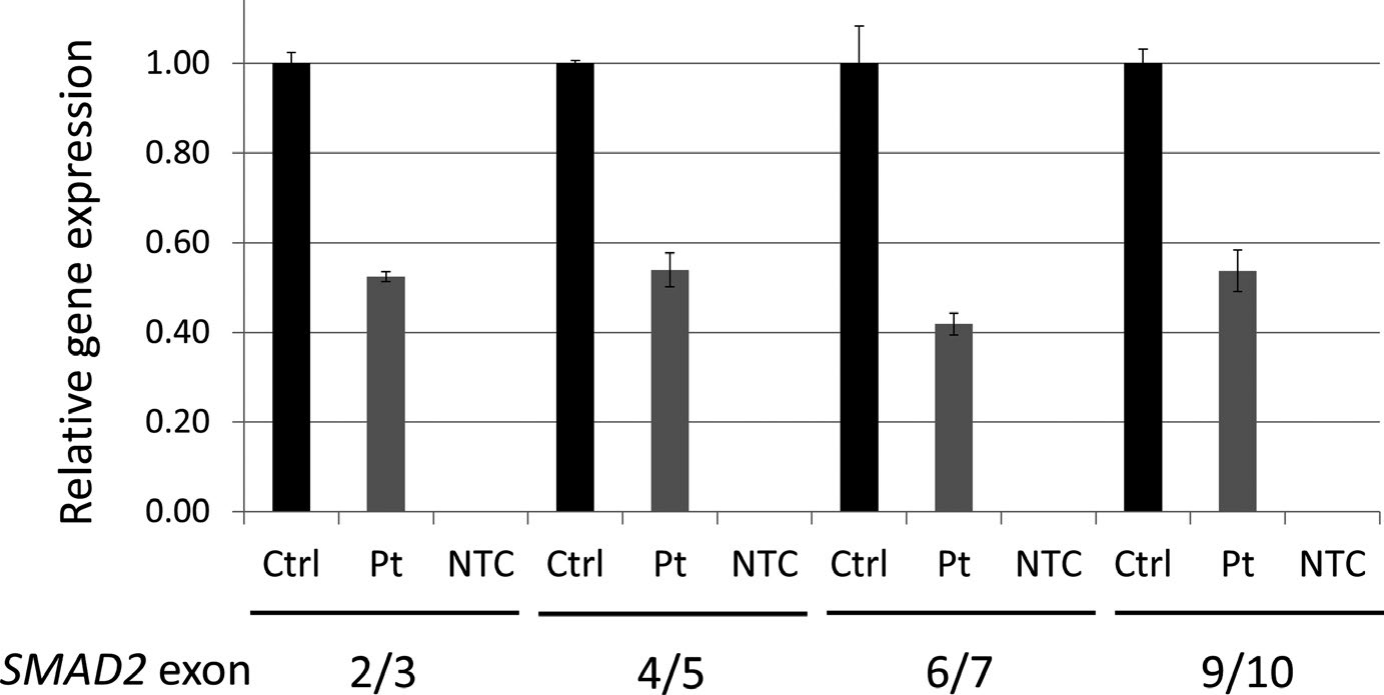
*SMAD2* RNA expression analysis. Total mRNA was isolated from cultured proband amniocytes, and RNA extracted from the amniocytes of a normal male with normal *SMAD2* copy number and with no phenotypic evidence of dextrocardia was used as a control. Real‐time RT‐PCR analysis targeted the *SMAD2* exon junctions 2/3, 4/5, 6/7, and 9/10 indicated underneath the black horizontal bars in both the proband (gray bars and Pt label) and the control (black bars and Ctrl label). NTC, no template control. The expression of *SMAD2* is significantly reduced in proband compared with the normal control

SMAD proteins regulate cell growth and differentiation by mediating TGF‐β signaling.^16^ In humans, heterozygous loss‐of‐function mutations of *SMAD2* have been linked to congenital heart disease, including dextrocardia^14^ and arterial aneurysms and dissections.^15^ While the *SMAD2* duplication has a highly significant genotype‐phenotype association in the proband (and subsequently affected sister), the interpretation of the duplication by CMA alone was uncertain given that CMA does not give positional information for the rearrangement (ie, the duplication could be located in direct or inverted orientation to the normal *SMAD2* allele, or it could be located elsewhere in the genome). To clarify the contribution of the *SMAD2* duplication in proband's phenotype, MPseq analysis was performed on fetal amniocytes. The duplication was mapped to chr18:47850720‐48005918 (GRCh38). PCR amplified the predicted MPseq junctions, and breakpoints are estimated to be located between chr18:47850012‐48005625 and chr18:47851237‐47852159 (GRCh38) (Figure S2). Structurally, the duplication was found to be in an inverted tandem orientation to the normal *SMAD2* allele, a pattern predicted to disrupt its coding sequence (Figure 2B,C and Figure S3). Gene expression analysis of the partial *SMAD2* duplication revealed *SMAD2* mRNA levels to be reduced by half compared with a normal male fetal amniocyte control, in agreement with the predicted *SMAD2* allele disruption (Figure 3).

## 3 DISCUSSION

Clinical interpretation of genomic duplications identified by CMA is a challenge faced by clinical genetic laboratories. As exemplified by this report, clarifying the genotype‐phenotype correlations of genomic duplications requires molecular breakpoint characterization since duplications can be pathogenic by more than one mechanism, including gene dosage alterations, gene disruption at breakpoints, creation of pathogenic gene fusions, and rewiring of the local and distal regulatory landscape of chromatin organization.^6^, ^17^

For the presented prenatal case, the 145‐Kbp duplication was found to be in an inverted tandem orientation to the normal *SMAD2* allele, disrupting the coding sequence of this gene and reducing *SMAD2* expression to about half of its normal value. The characterization of this duplication and its associated functional impact could not have been achieved without a molecular method, such as MPseq, to determine rearrangement positional information. Similar to our case, MPseq has been applied to the analysis of structural variants in the prenatal setting, and shown to resolve complex chromosome rearrangements with important consequences to prenatal diagnosis and perinatal management.^18^ To our knowledge, this is the first report of a partial *SMAD2* duplication in an inverted orientation associated with heterotaxy. In a murine model, *Smad2* has been shown to act in a dosage‐dependent manner in the Nodal signaling pathway to regulate left‐right developmental patterning.^13^, ^19^ Similarly in humans, *SMAD2* has been classified as a haploinsufficient gene as evidenced by reports of deletions of *SMAD2* in association with congenital heart defects, including dextrocardia.^14^

It remains uncertain how the presence of the duplication in the proband's mother was not tied to any major clinical concerns despite the inheritance of the duplication in affected offspring. While duplications are often parentally inherited, the functional outcome and interpretation can be obscured by mosaicism, incomplete penetrance, and/or variable expressivity. For example, a subject with a parentally inherited 2.13‐Mbp deletion encompassing *SMAD2* reported in the Database of Chromosomal Imbalance and Phenotype in Humans using Ensembl Resources (DECIPHER)^20^ does not present with congenital heart defects, but pulmonary stenosis was detected, a feature that can also be seen in heterotaxy (Table S2). In contrast, ClinVar^21^ entries of *SMAD2* mutations include four cases with reported cardiac anomalies (Table S3).^14^ For M1, we were unable to determine whether the partial *SMAD2* duplication was mosaic, or whether additional heterotaxy‐related pathogenic variants were present in her genome or that of her partners.

With more cases being published and variants made accessible to the genetics community through collective efforts such as the DECIPHER and ClinVar databases, prediction of clinical outcomes of prenatal duplications may be more accurate. Nucleotide‐level resolution studies will, ultimately, provide the final answer to the complex problem of clinical duplication interpretation in routine genetic testing.

## 4 MATERIALS AND METHODS

Genetic testing was performed on an amniocentesis sample from M1. Aneuploidy FISH analysis was performed on 100 nuclei from uncultured amniocytes and tested chromosomes X, Y, 13, 18, and 21. CMA was performed on cultured amniocytes using the Affymetrix CytoScan HD platform (Thermo Fisher Scientific, Waltham, MA). MPseq was performed on DNA extracted from amniocytes using AutoPure LS (Qiagen, Hilden, Germany). MPseq libraries were prepared using Nextera Mate Pair Library Preparation Kit (Illumina, San Diego, CA), which were subsequently purified and used for short‐read library preparation using TruSeq DNA Library Prep kit (Illumina, San Diego, CA). Purified libraries were sequenced in an Illumina HiSeq 2500 using RapidRun mode to obtain 101‐bp reads. MPseq data were processed with BIMAv3^10^ and analyzed with SVAtools version 0.24.9.^8^ Polymerase chain reaction (PCR) experiments were performed to amplify breakpoint junctions identified by MPseq. Gene expression experiments were performed on total mRNA purified from cultured amniocytes of the proband and a normal control male with normal *SMAD2* copy number and no phenotypic evidence of dextrocardia. Relative mRNA levels were determined by real‐time RT‐PCR analysis using Applied Biosystems TaqMan Assays (Thermo Fisher Scientific) targeting the *SMAD2* exon junctions 2/3, 4/5, 6/7, and 9/10. Three technical replicates were used to calculate standard deviation for both the proband and control RNA expression (primers used are listed in Table S1).

## CONFLICT OF INTEREST

None declared.

## AUTHOR CONTRIBUTIONS

LBB and HMK: conceptualized the study. CJZM, AE, SAS, SHJ, NLH, HMK, and LBB: performed formal analysis. CJZM and LBB: wrote the manuscript. DLJ, GV, and LBB: collected resources.

## Funding information

This study was supported by the Mayo Clinic Department of Laboratory Medicine and Pathology.

## ETHICAL APPROVAL

Approval for this study was received by the Mayo Clinic Institutional Review Board, application number 16‐002365; participants were consented accordingly for release of data included in this study.

## Supporting information

Fig S1‐S3

Tab S1‐S3

## Data Availability

The data that support the findings of this study are available in the supplementary material of this article.
