# Supplementary material for: Prenatal characterization of a novel inverted SMAD2 duplication by mate pair sequencing in a fetus with dextrocardia
Source: Clin Case Rep. 2020 Dec 10;9(2):769–74. doi: 10.1002/ccr3.3608 (PMC7869371; doi:10.1002/ccr3.3608)
Supplement: Supplementary file 1 — Fig S1‐S3 [file CCR3-9-769-s001.docx]

A)

D13, D21

DXZ1, DYZ3, D18Z1


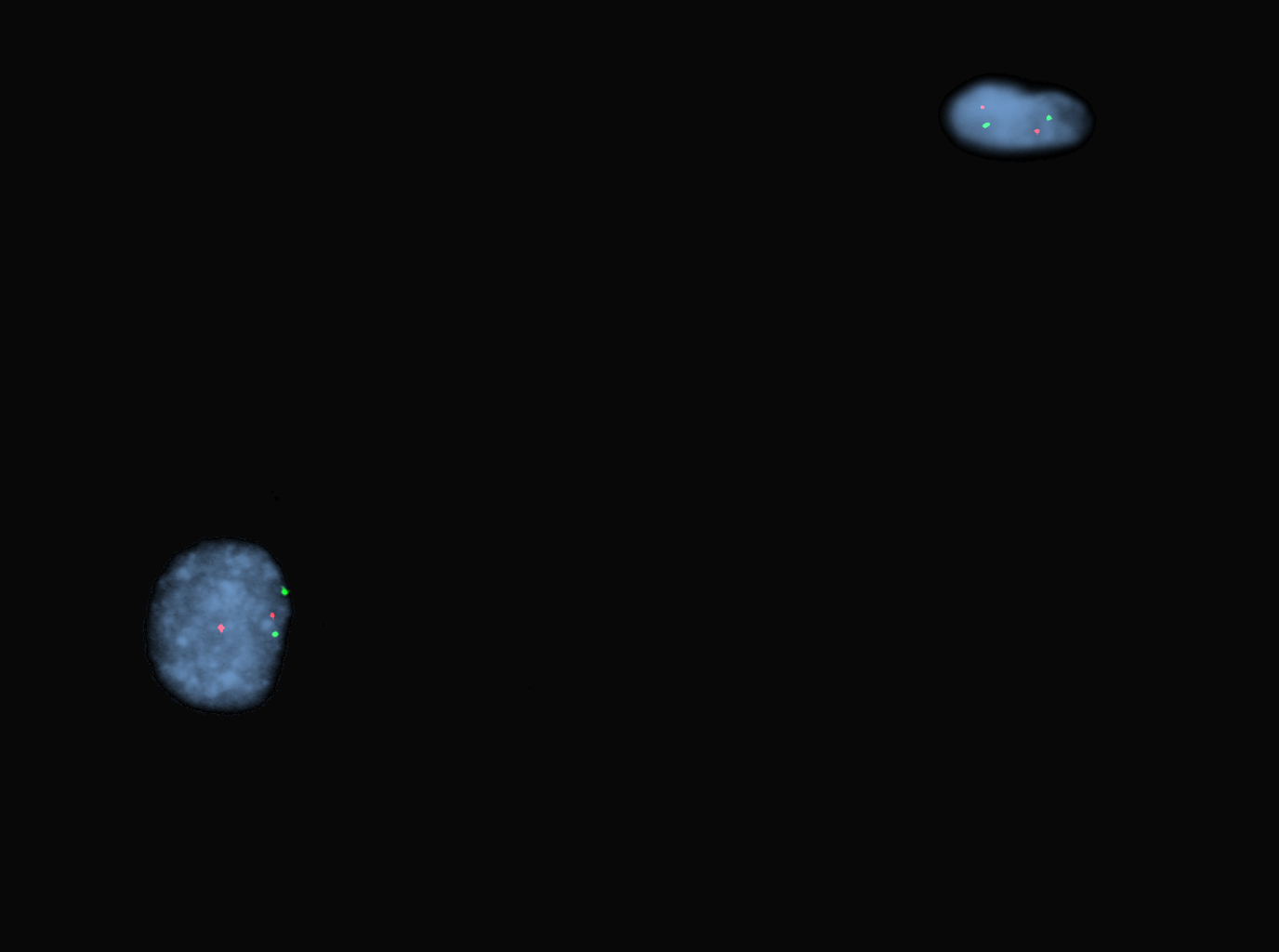

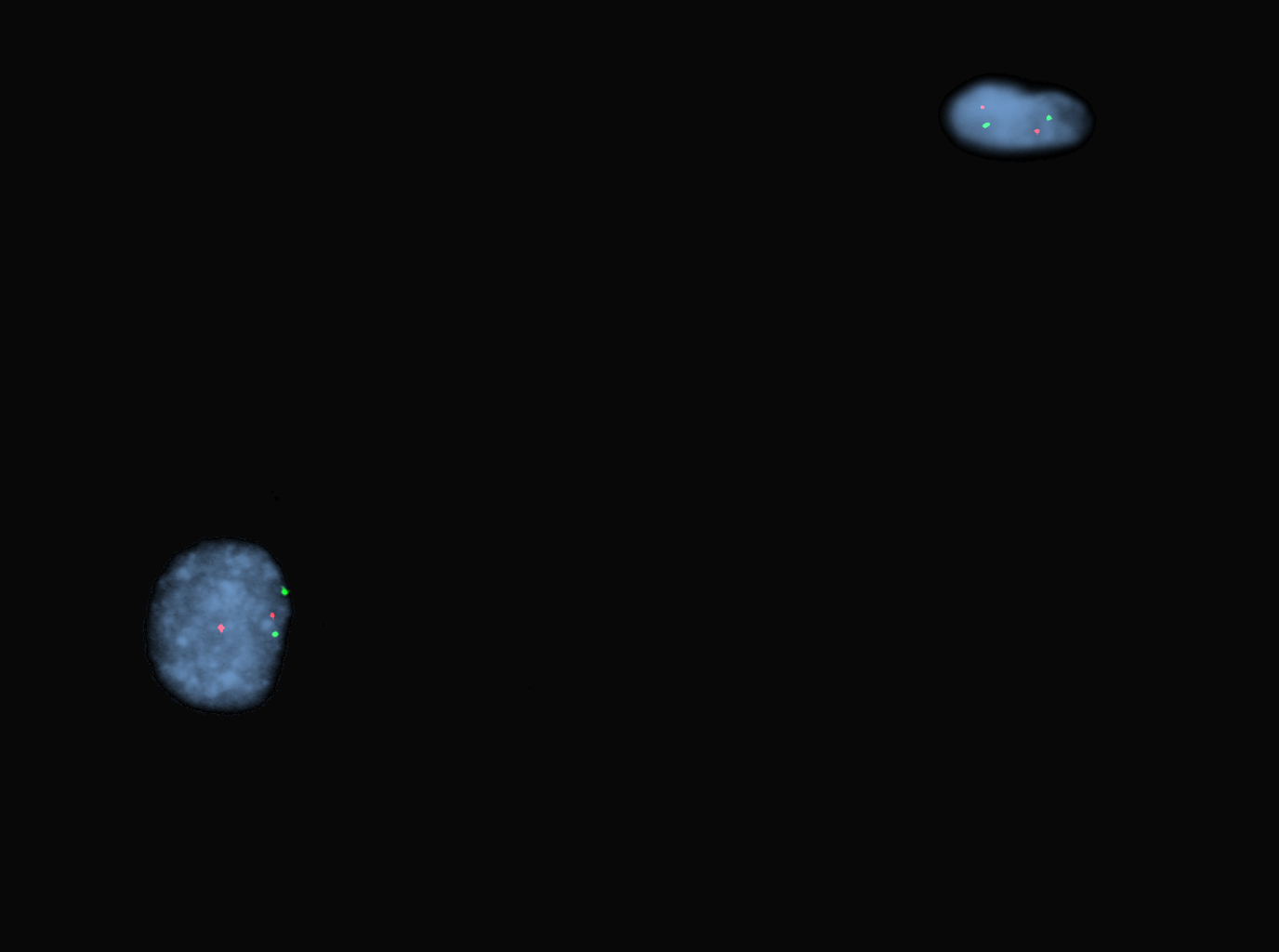

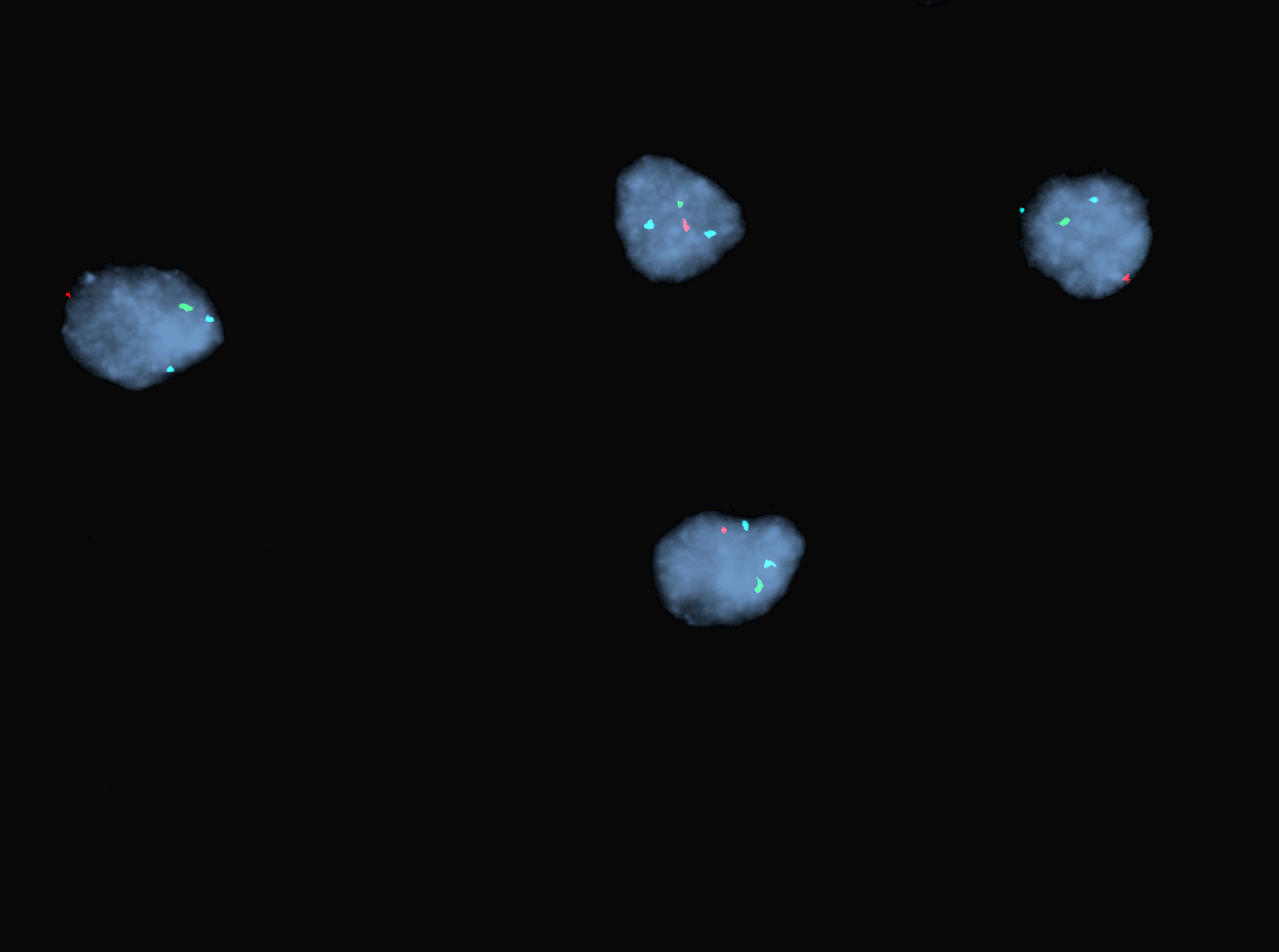

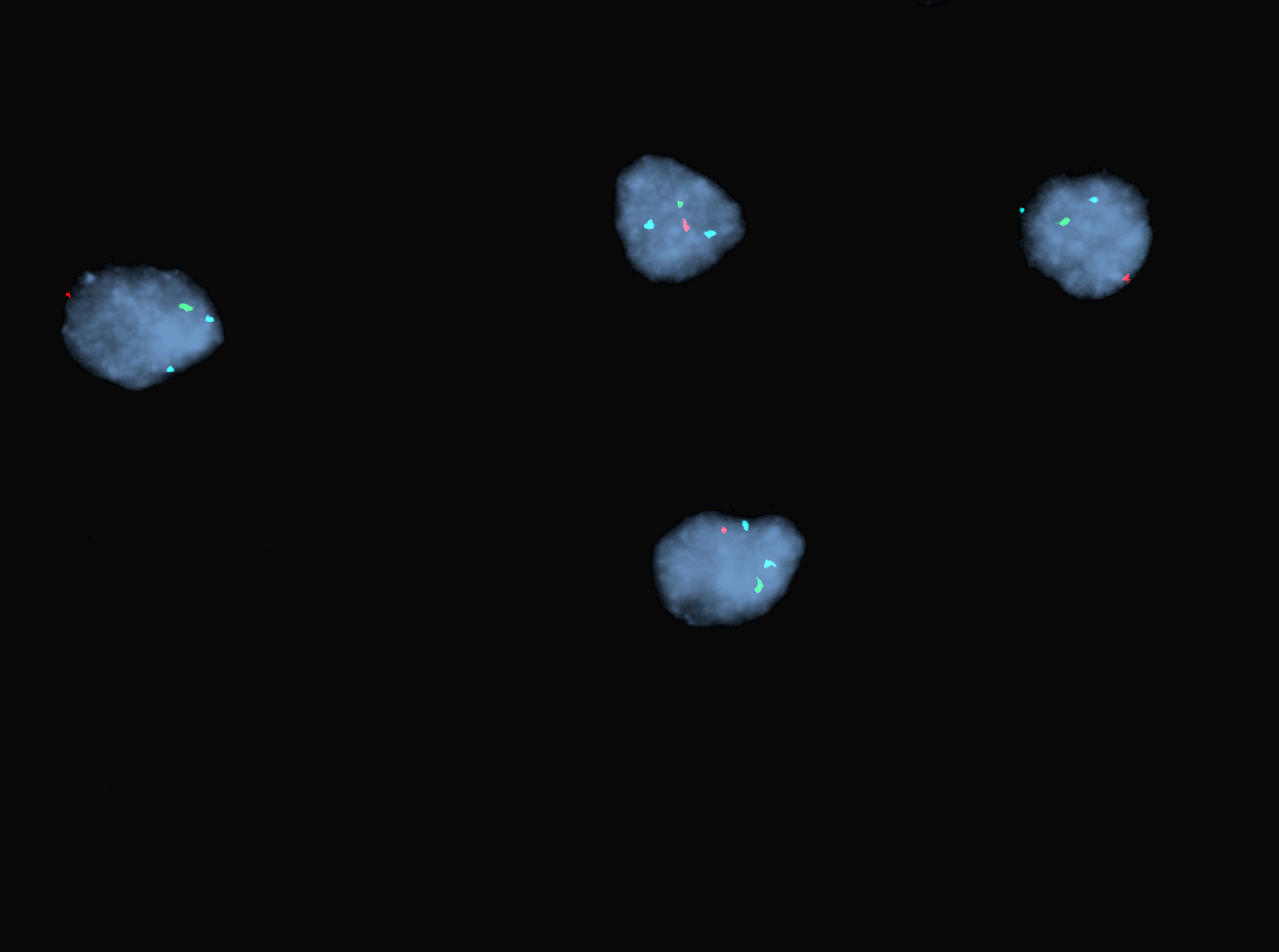


B)

Supplementary Figure 1. Representative images for aneuploidy FISH analysis on 100 nuclei from uncultured amniocytes. A) FISH probes for chromosomes X, Y, and 18 are color-coded in green, red, and aqua, respectively. B) FISH probes for chromosomes 13 and 21 are shown in green and red, respectively. Nuclear DAPI-staining is shown in blue.


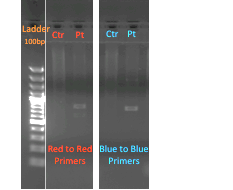


Supplemental Figure 2. PCR amplification of red-red and blue-blue junctions used for sequencing the duplication breakpoints. Breakpoints were estimated to be at chr18:47850012- 48005625 and chr18:47851237- 47852159 (hg38).


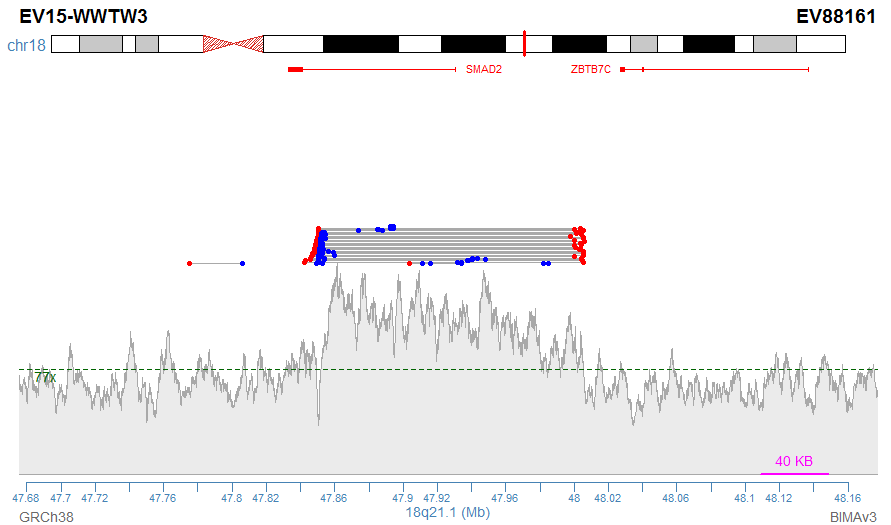


146Kb Duplication

Supplemental Figure 3. The SMAD2 rearrangement as seen in our MPseq analysis pipeline. Chromosome 18 is shown and the vertical red line represents the location of the duplication. Gene positions are shown in red with their corresponding names. Colored dots joined by black lines indicate discordant MPseq reads mapping >15 kb apart. Blue dots correspond to forward strand mapping reads and red dots are reads mapping to the reverse strand. The histogram represents read mapping for CNV analysis. The ~145Kb duplication is seen as an increase above the 2n (green line) level.
